# Iterative reconstruction improves detection of in-stent restenosis by high-pitch dual-source coronary CT angiography

**DOI:** 10.1038/s41598-017-07499-9

**Published:** 2017-07-31

**Authors:** Junjie Yang, Xiaobo Yang, Carlo N. De Cecco, Taylor M. Duguay, Zhiye Chen, Christian Tesche, U. Joseph Schoepf, Yundai Chen

**Affiliations:** 1 Department of Cardiology, PLA General Hospital, Beijing, China; 2 Division of Cardiovascular Imaging, Department of Radiology and Radiological Science, Medical University of South Carolina, Charleston, SC USA; 3 Department of Radiology, PLA General Hospital, Beijing, China

## Abstract

Recent studies demonstrated that sinogram affirmed iterative reconstructions (SAFIRE) can produce higher-resolution images with greater robustness for the reduction of various imaging artefacts. Eighty-five patients were prospectively evaluated and underwent a high-pitch spiral acquisition CT scan. In-stent noise, signal-to-noise ratio(SNR), stent-lumen attenuation increase ratio (SAIR), and subjective image quality score were measured and compared between the SAFIRE and Filter back projection (FBP) reconstructions. Conventional coronary angiography served as the standard of reference. In 159 evaluated stents, SAFIRE was superior to FBP with regards to in-stent noise, SNR, SAIR, and image quality score. On per-stent analysis, SAFIRE vs. FBP reconstruction yielded 85% vs. 85%sensitivity, 89% vs. 78%specificity, 73% vs. 57%positive predictive value, 95% vs. 94%negative predictive value, and 0.87 vs. 0.82 area under curve, although these improvements did not reach statistical significance (P > 0.05). However, in the subgroup of small diameter stents (≤3 mm; n = 95), specificity(82% vs. 62%), positive predictive value(66% vs. 50%) and area under curve (0.81 vs. 0.70) improved significantly (P < 0.05) with SAFIRE. SAFIRE image reconstruction can thus improve the evaluation for ISR, especially in smaller stents.

## Introduction

Conventional coronary angiography (CCA) remains the diagnostic standard for evaluating in-stent patency for the follow-up assessment of percutaneous coronary stenting. With the substantial improvement of both temporal and spatial resolution, coronary computed tomography angiography (CCTA) is becoming a reliable noninvasive test for surveillance and detection of in-stent restenosis (ISR)^1^. However, metal-related image artefacts are a known limitation of CCTA and may impede appropriate visualization of the stent lumen and quantification of lumen narrowing^2^. Guidelines state that it is reasonable to evaluate stents greater than 3 mm in diameter with CCTA, but the performance of CCTA decreases dramatically with stents less than 3 mm in diameter^3^.

Additionally, concerns remain regarding the radiation exposure from CCTA. For low-dose CCTA, prospectively electrocardiogram (ECG)-triggered acquisition has been widely introduced and generally accepted^4^. Recently, prospectively ECG-triggered single heart-beat high-pitch spiral acquisition dual source CT (DSCT) has become a viable alternative to other ECG-synchronization methods^5, 6^. Our previous study demonstrated that the high-pitch mode maintains diagnostic accuracy for the assessment of significant ISR and significantly lowers the radiation dose^7^. However, the reduction in radiation dose results in an increase in image noise compared to traditional cardiac CT acquisition methods.

Recently, iterative reconstruction (IR) techniques are replacing filtered back projection (FBP) as the default image reconstruction method in CCTA. In particular, it has been demonstrated that sinogram affirmed iterative reconstruction (SAFIRE) can produce higher fidelity images with greater robustness for the reduction of various imaging artefacts by reducing image noise without affecting spatial resolution^8–10^. However, insufficient data exists quantifying the potential gains in diagnostic accuracy for ISR detection, especially in small stents.

The aim of this study was to evaluate the performance of SAFIRE for the detection of ISR using second generation DSCT with a prospectively ECG-triggered high-pitch spiral acquisition in symptomatic stent-recipients referred for CCA.

## Results

### Baseline characteristics

Of the 109 patients screened for inclusion, 24 were excluded due to impaired renal function (n = 4), contraindication to contrast agents (n = 3), irregular heart rate (n = 9),or heart rate > 65 bpm despite β-blockade treatment (n = 8). A total of 85 patients underwent HPS-DSCT.

Patients’ baseline clinical parameters are listed in Table 1. The average age of included patients was 64.5 ± 16(range: 41–73) years.

**Table 1:** Patients’ clinical parameters.

Characteristics	Data (n = 85)
Age (yrs)	64.5 ± 16
Interval between stent and inclusion (months)	22 ± 13
Male (n, %)	50 (59%)
Body mass index (kg/m^2^)	25 ± 5
Diabetes mellitus (n, %)	50 (59%)
Hypertension (n, %)	71 (84%)
Hypercholesterolemia (n, %)	62 (73%)
Current smoker (n, %)	29 (34%)
Family history (n, %)	33 (39%)

Values are n, mean ± SD or n (%).

### Findings related to coronary artery stents

We examined 126 stented lesions (159 stents used, 1.87 stents per patient). Nighty-Three lesions consisted of single stents; the remaining 33 lesions consisted of overlapping stents (n = 23) and bifurcations (n = 10), also referred to as complex configuration stenting. Bare metal stents (BMSs) accounted for 14.5% (23/159) of the stented lesions and drug-eluting stents (DESs) were used in 85.5% (136/159). Restenosis was diagnosed by CCA in 41 out of 159 (25.8%) stents and in 24 out of 85 (28.2%) patients. Restenosis was found in 11 out of 23 (47.8%) BMSs and in 30 out of 136 (22.1%) DESs. In the simple and complex configuration intervention subgroups, frequency of restenosis was 26.9% (25/93) and 48.5% (16/33), respectively. In the stent diameter subgroups (>3 mm and ≤3 mm), frequencies of ISR were 18.8% (12/64) and 30.5% (29/95), respectively (p < 0.05). The mean strut thickness in the included stents was 110.5 ± 22.1 µm. The mean stent diameter and the mean stent length in the entire sample were 3.0 ± 0.4 mm and 21.4 ± 8.3 mm, respectively. Frequencies of restenosis were 18.9% (30/159) in the inner-stent and 6.9% (11/159) in the peri-stent area, respectively (p < 0.05). Image acquisition parameters and stent characteristics are summarized in Tables 2 and 3.

**Table 2:** Image acquisition parameters and stent characteristics.

Scan Parameters	
Tube potential (kVp)	100
Tube current (mAs)	273 ± 37
CT dose index volume (mGy)	5.8 ± 1.9
Z-coverage (cm)	15.5 ± 3.7
Dose-length product (mGy × cm)	89.1 ± 16.7
Estimated effective dose (mSv)	1.4 ± 0.5
Stent Characteristics	
No. of stents	159
Mean No. of stents per patient	1.87
Mean stents’ diameters, mm	2.9 ± 0.6
No. of DESs	119 (86.9%)
No. of complex configuration	76 (47.8%)
No. of small stents (≤3 mm)	95 (59.7%)
Mean stent length, mm	21.3 ± 8.1
Mean stent strut thickness, µm	110.6 ± 21.1

Values are n, mean ± SD or n (%). DESs equaled Drug-Eluting Stents. Complex configuration meant overlapping or bifurcation stenting procedure.

**Table 3:** Detailed information for included stents.

Stent name	n	Strut thickness (μm)	Diameter (mm)	Length (mm)
Cypher^®^ (Cordis, Miami, USA)	25	140	2.9 ± 0.3	23.8 ± 5.2
Endeavor^®^				
(Medtronic,Minneapolis, USA)	28	97	2.8 ± 0.4	21.2 ± 8.4
Partner^®^ (Lepu, Beijing, China)	30	114	3.0 ± 0.5	22.6 ± 9.7
Firebird (Microport,Shanghai, China)	40	100	2.8 ± 0.4	19.6 ± 7.6
Others^*^	36	113.2 ± 42.4	3.1 ± 0.5	20.1 ± 9.1
Total	159	110.5 ± 22.1	3.0 ± 0.4	21.4 ± 8.3

*Taxus®(Boston Scientific, Natick, USA) n = 6, Excel® (JW Medical System, Shandong, China) n = 7, Lepu BMS®(Lepu) n = 9, Driver® (Medtronic) n = 2, Penta® (Guidant, Indianapolis USA) n = 2, Titan® (Hexacath, Paris, France) n = 5, Zeta® (Guidant) n = 4, Jostent Flex® (JOMED AB, Helsingborg, Sweden) n = 1.

### Subjective and objective image quality

Image quality parameters of SAFIRE were significantly better compared with traditional FBP both subjectively and objectively **(**Table 3). With SAFIRE, the subjective image quality score increased from 2.4 ± 1.1 points to 3.1 ± 0.7 points (p < 0.05). In addition to significantly lower in-stent image noise with SAFIRE as compared to FBP, there was a significantly higher contrast-to-noise ratio with the former technique**(**Table 4 **)**. Furthermore, the stent lumen attenuation increase ratio (SAIR) was significantly lower with SAFIRE compared to the FBP reconstruction. This improvement was due to a substantial reduction in image noise by SAFIRE compared to FBP (73.2 ± 17.0 HU for SAFIRE vs. 108.9 ± 22.1 HU for FBP; p < 0.001).

**Table 4:** Evaluability performance of HPS-DSCT by SAFIRE.

	SAFIRE(n = 159)	FBP(n = 159)	*P* value
In-stent noise	73.2 ± 17.0	108.9 ± 22.1	<0.001
SNR	21.0 ± 7.9	15.7 ± 6.2	<0.05
SAIR	22.9 ± 12.8	36.4 ± 24.1	<0.05
Subjective image quality	3.1 ± 0.7	2.4 ± 1.1	<0.05
Non-diagnostic (n, %)	4(2.5%)	9 (5.7%)	<0.05

SNR = in-stent signal to noise ratio. SAIR = stent lumen attenuation increase ratio.

### Diagnostic performance of DSCT

All 159 stents were detected by CCTA. Altogether, 4 stents (2.5%) after SAFIRE and 9 stents (5.7%) after FBP were rated non-diagnostic and were therefore rendered as having significant in-stent restenosis for further statistical analysis.

**Figure 1:**
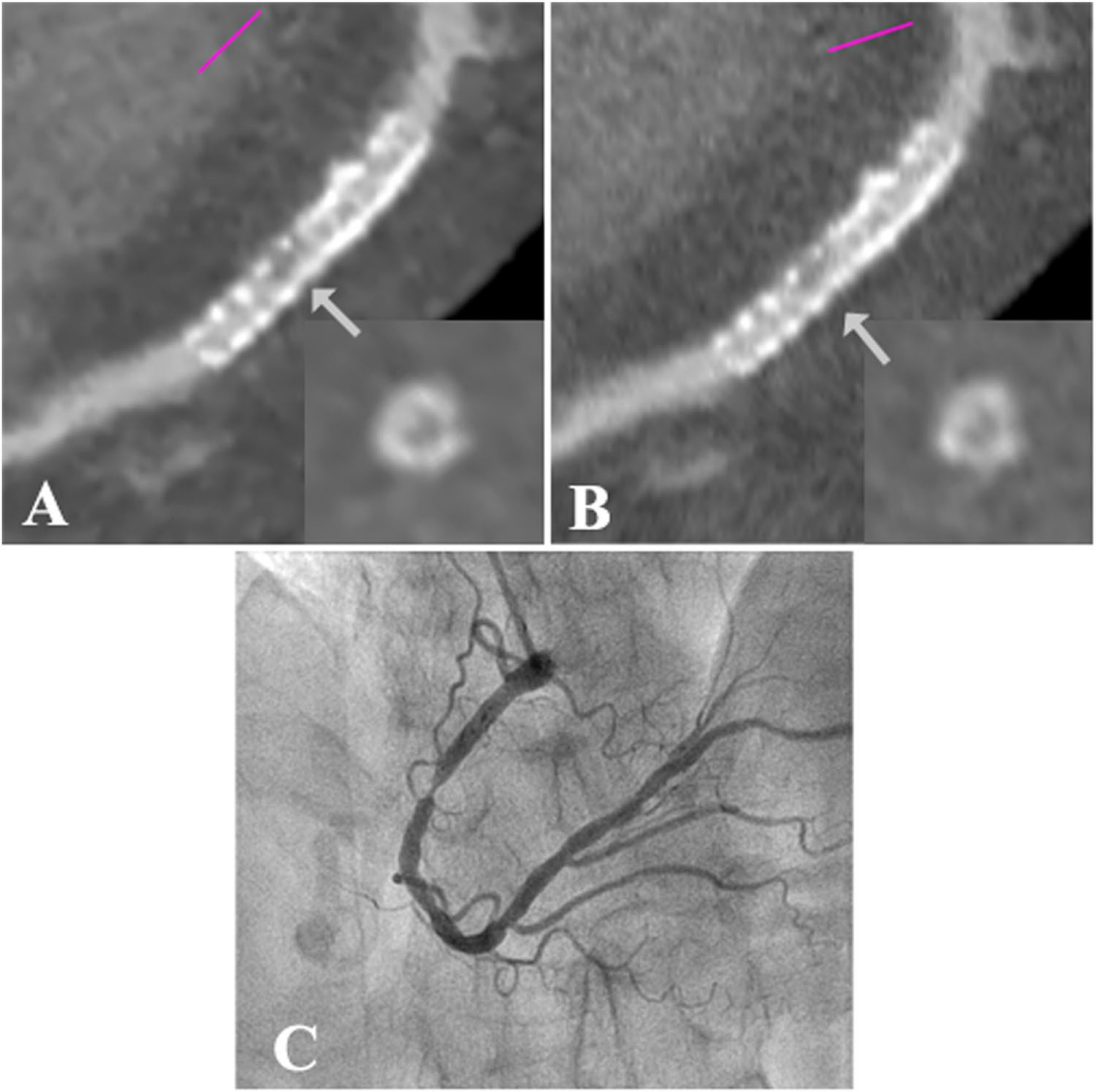
Example of the presence of in-stent restenosis(3.0 mm Diameter Drug-Eluting Stent). (**A**) Sinogram affirmed iterative reconstruction dataset with curved multi-planar reconstruction of the right coronary artery (RCA). Arrow indicates the presence of significant in-stent restenosis. Boxed area clarifies the cross-section view. (**B**) Filtered back projection dataset with curved multi-planar reconstruction of RCA. Arrow indicates the presence of significant in-stent restenosis. Boxed area clarifies the cross-section view. (**C**) Corresponding conventional coronary angiogram showed significant luminal narrowing (plus 50%) of in-stent patency in the mid part of RCA.

**Figure 2:**
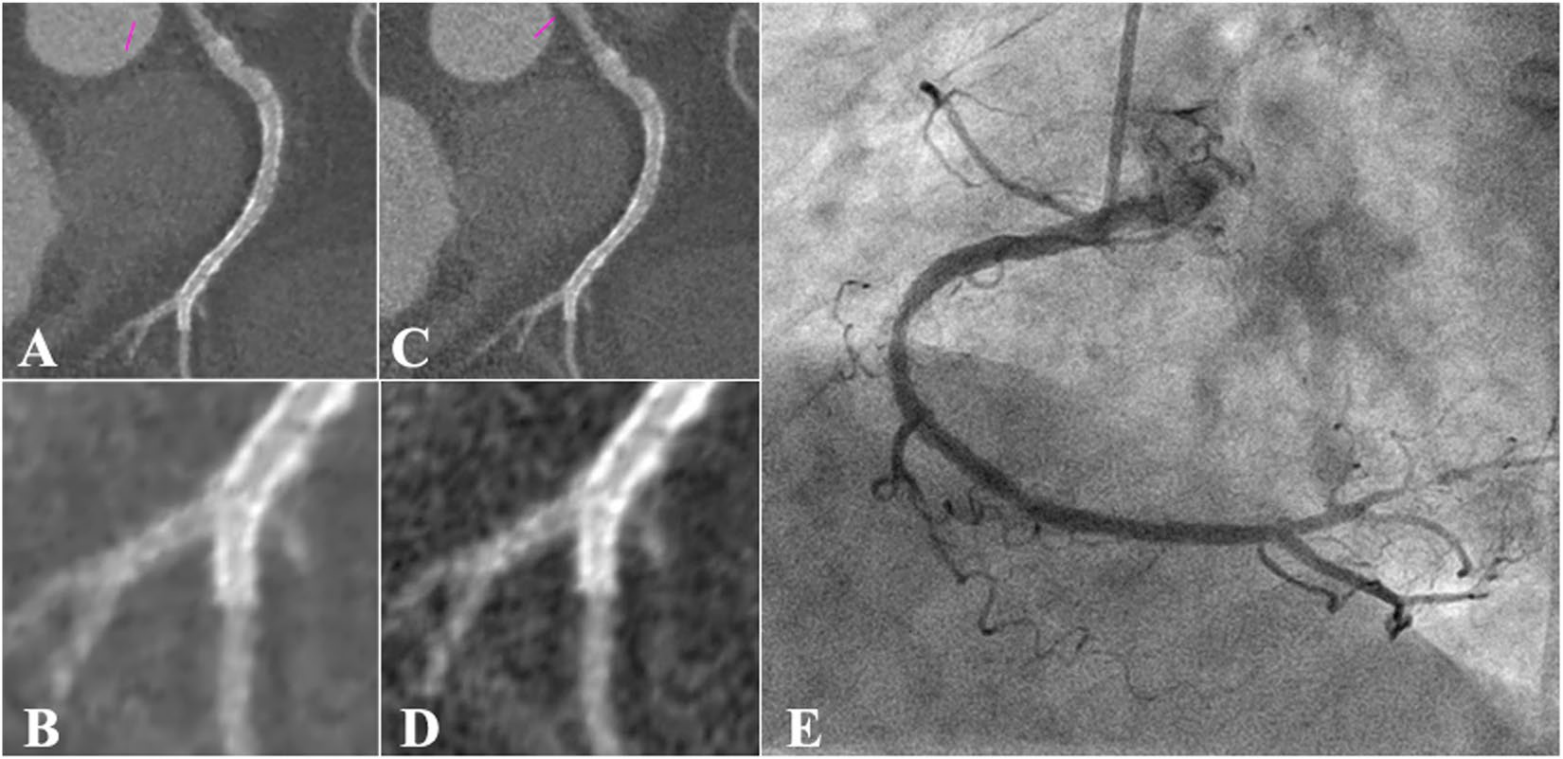
Example of the absence of in-Stent restenosis (2.75 mm Diameter Drug-Eluting Stent). **(A)**Sinogram affirmed iterative reconstruction dataset with curved multi-planar reconstruction of the RCA. It showed no significant in-stent restenosis especially in magnified scale **(B**,**C**) Filtered back projection dataset with curved multi-planar reconstruction of the RCA. It showed uncertain in-stent patency even in magnified scale (**D**,**E**) Corresponding conventional coronary angiogram confirmed the absence of significant luminal narrowing within the stents.

In total, 41 out of 159 stents showed ISR by CCA. Thirty-five of the 41 stents with ISR were correctly identified with SAFIRE reconstruction as well as FBP reconstruction **(**Fig. 1), resulting in sensitivity, specificity, PPV, NPV and AUC of 85%, 89%, 73%, 95% and 0.87, respectively, with SAFIRE, and 85%, 78%, 57%, 94% and 0.82, respectively, with FBP for detecting ISR. As a result, there was a trend towards improvement without reaching statistical significance in the entire sample stents (P = 0.10), as well as in stents with a diameter greater than 3 mm (P = 0.39). However, the diagnostic performances obtained in stents with a diameter less than 3 mm favored the SAFIRE reconstruction **(**Fig. 2 **)**. Here, sensitivity, specificity, PPV, and NPV were 79%, 82%, 66%, and 90%, respectively with SAFIRE, versus 79%, 62%, 50% and 86% with FBP. Compared to FBP reconstruction the AUC was thus significantly improved with SAFIRE [0.81 (0.71, 0.88) vs. 0.70(0.60, 0.79), p = 0.03 in Table 5] in the small stent subgroup (≤3 mm).

**Table 5:** Diagnostic performance of iterative reconstruction in detecting in-stent patency.

	Total stent (N = 159)		>3 mm stents (N = 64)		≤3 mm stents (N = 95)	
	SAFIRE	FBP	SAFIRE	FBP	SAFIRE	FBP
Total	159	159	64	64	95	95
TP	35	35	12	12	23	23
TN	105	92	51	52	54	38
FP	13	26	1	0	12	26
FN	6	6	0	0	6	6
Sensitivity	0.85 (0.72,0.93)	0.85 (0.72,0.93)	1.00 (0.75,1.00)	1.00 (0.76,1.00)	0.79 (0.61,0.90)	0.79 (0.61,0.90)
Specificity	0.89 (0.82,0.93)	0.78 (0.70,0.85)	0.98 (0.90,1.00)	1.00 (0.93,1.00)	0.82 (0.71,0.89)	0.62 (0.50,0.73)
PPV	0.73 (0.59,0.83)	0.57 (0.45,0.69)	0.92 (0.67,1.00)	1.00 (0.76,1.00)	0.66 (0.49,0.79)	0.50 (0.36,0.64)
NPV	0.95 (0.89,0.98)	0.94 (0.87,0.98)	1.00 (0.93,1.00)	1.00 (0.93,1.00)	0.90 (0.80,0.95)	0.86 (0.73,0.94)
AUC	0.87 (0.81,0.92)	0.82 (0.75,0.87)	0.99 (0.93,1.00)	1.00 (0.94,1.00)	0.81 (0.71,0.88)	0.70 (0.60,0.79)

Values are n or n/N[95% CI]. FBP = Filtered back projection. SAFIRE = sinogram affirmed iterative reconstruction. TP = True Positive. TN = True Negative. FP = False Positive. FN = False Negative. NPV = Negative Predictive Value; PPV = Positive Predictive Value; AUC = Area Under Curve.

A reproducibility analysis was done for the two readers. Inter-observer agreement for detecting restenosis was good (k-value = 0.78), while intra-observer agreement was excellent (k-value = 0.86).

### Radiation dose and contrast medium volume

The mean tube current–time product/tube voltage, average CTDI volume, and scan length were 273 ± 37 mAs/100 kV, 5.8 ± 1.9 mGy and 15.5 ± 3.7 cm, respectively. The average DLP and effective radiation dose equivalent were 89.1 ± 16.7mGycm and 1.4 ± 0.5 mSv. A mean volume of (67 ± 9)mL of contrast medium was administered.

## Discussion

The aim of this study was to evaluate the comparative performance of DSCT in high-pitch mode with SAFIRE reconstruction for evaluating coronary stent patency, using CCA as a reference standard. In addition, we also investigated the effect of this technique on image quality, both objectively and subjectively. To our knowledge, the benefits of combining high-pitch spiral acquisition and iterative reconstruction has not been previously investigated. Also, it is important to note that small-size stents (diameters less than 3 mm) were included in this study and analyzed as a subgroup. The present study found that SAFIRE can improve the evaluation of coronary artery stents using DSCT compared to traditional FBP, with a significantly improved diagnostic performance in small-sized stents. Several previous results had demonstrated the reliability of coronary CTA to rule out in-stent restenosis especially in larger stents, but the coronary CTA remained problematic and should not be recommended on a general basis in stent with smaller diameters. For small stent, SAFIRE in the present study showed comparable sensitivity and negative predictive value, with statistically significant, higher specificity, positive predictive value, area under ROC curve and number of assessable stents.

The most common factor that limits the CT evaluation of stents is “blooming artefact” arising from metallic stent struts^11^. Stent blooming is caused by beam-hardening, which in turn can cause artefactual hypo-attenuation near the stent meshes^12^. These artefacts scan trigger a false positive finding of ISR or overestimation of lesion severity, especially in-stents containing denser metal alloys and/or stents with diameters smaller than 3.0 mm.

In this investigation, SAFIRE showed superior image quality over FBP both subjectively and objectively, which is in line with previous studies^13^. Also, there were fewer non-diagnostic stents with SAFIRE(2.5% vs. 5.7% with FBP, P < 0.05), likely as a result of the reduction in image noise. Comparing the stent lumen attenuation increase ratio, SAFIRE significantly reduced the in-stent image noise and lowered the metal-related attenuation increment, resulting in improved in-stent visualization. However, compared to a previous SAFIRE study^12^, the in-stent image noise observed in our study was higher, which is most likely due to the different scan modes used in the studies. In fact, there was only one case of a high-pitch spiral acquisition in this previous study. Like other studies, the SNR correlated inversely with the SAIR, which showed objective reduction in metal related image noise^14, 15^.

The improved image quality of SAFIRE led to an overall superior AUC compared to FBP (0.87 vs. 0.82); however, this strong trend did not reach statistical significance (p = 0.10). Conversely, in stents with a diameter less than 3 mm, SAFIRE showed a statistically significant improvement in specificity (0.82 vs. 0.62), positive predictive value (0.66 vs. 0.50) and AUC (0.81 vs. 0.70) compared to FBP. One meta-analysis showed moderate specificity (0.85) and limited sensitivity (0.95) for determining in-stent patency by CCTA^16^, while most of the included studies excluded small stents. A recent study also showed that almost 20% of the enrolled patients, mostly with small stents, were unsuccessfully imaged with FBP^17^.

The advantages of iterative reconstruction techniques for CCTA for the reduction of CT image noise have been studied in coronary stents *in vitro* and *in vivo* and are well recognized^18^. In detail, SAFIRE greatly reduced image noise related to particular factors known to negatively influence the performance of CCTA in stent evaluation; such as stent size, strut thickness, and overlapping stents^2^.

Our previous study indicated that DSCT in high-pitch mode with traditional FBP provided good diagnostic accuracy of coronary stent patency compared to CCA. However, the increased image noise, though not statistically significant, posed limitations for the assessment of small stents. Combined with SAFIRE, which enhances the low-contrast detectability and improves streak artefacts^19^, high-pitch spiral acquisition could provide an appropriate approach for the evaluation of in-stent patency. The relatively low radiation exposure could ultimately benefit follow-up assessment of in-stent patency by CCTA.

DSCT appears advantageous, due to its high temporal resolution, increased acquisition pitch, shorter scan time, and lower radiation dose^20^. High-pitch spiral acquisition scan reduce radiation exposure by more than 50% compared to prospective sequential acquisitions^21^. Studies comparing radiation doses with DSCT have reported even more substantial dose reduction than in the present study, including comparisons against retrospective gating spiral protocols as well as prospective triggering sequential mode^22, 23^. This study has demonstrated the superiority of a prospectively triggered high-pitch acquisition mode. In fact, even though Eisentopf *et al*.^14^, achieved a lower dose (below 1 mSv) using sequential acquisition combined with iterative reconstruction,25% of small stents were not evaluable in this previous study. This may be in part due to the fact that sequential scanning of patients with an irregular heart rate can cause stair-step artefacts and can limit the accuracy of CCTA, which does not occur with a spiral acquisition mode.

This investigation has certain limitations that need to be considered. Firstly, although there are several studies that show improved image quality and radiation dose reduction with iterative reconstruction, quantitative data on clinical benefits and patient outcomes derived from the use of iterative reconstruction is still very scarce or absent. Secondly, we did not compare SAFIRE with DSCT in high-pitch mode with other image acquisition techniques to establish superiority of this scan mode. Finally, although our population is one of the larger ones published to date, the sample size may still be too small for accuracy and granular assessment of all effects.

In summary, SAFIRE reconstruction provides superior image quality that is beneficial for stent surveillance by low radiation-dose CCTA. In combination with SAFIRE image reconstruction, high-pitch spiral acquisition can thus aid the evaluation for ISR, especially in smaller stents.

## Methods

### Study population and procedures

Ninety-two patients with prior coronary stent implantation scheduled for CCA between September 2013 and March 2015 due to chest discomfort and/or a positive stress test were considered for inclusion in this study. Exclusion criteria consisted of: contraindications to iodinated contrast agents, impaired renal function (estimated Glomerular Filtration Rate < 60 mL/min), heart rate (HR) > 65 beats per minute (bpm) despite β-blocker administration, and cardiac arrhythmias. All included patients underwent CCA within 14 days after the CCTA scan. The mean interval between the initial percutaneous coronary intervention and CT examination was 22 months (range 13 to 69, median 25). The study protocol was approved by the institutional ethics review board of Chinese PLA general hospital and written informed consent was obtained from all patients. The patients’ clinical information (gender, age, date of previous stent implantation and DSCT, body height and weight) and stent parameters (type, material, diameter, length, strut thickness) were obtained via chart review. All methods were performed in accordance with the relevant guidelines and regulations.

### Imaging protocol

All examinations were performed on a 2^nd^ generation DSCT scanner (Definition Flash, Siemens Healthcare, Forchheim, Germany). Data acquisition was performed with a detector collimation of 2 mm × 64 mm × 0.6 mm, z-axis flying focus technique and gantry rotation of 280 ms. Patients with a body mass index(BMI) ≥ 26 kg/m^2^ were examined with a tube voltage of 120 kVp, whereas patients with a BMI < 26 kg/m^2^ with a tube voltage of 100 kVp. Each tube provided a maximum of 430 mAs/rotation.

Contrast agent (Ultravist 370, Schering, Berlin, Germany) was injected into the antecubital vein at a flow rate of 5.0 mL/s, followed by a saline chaser (40 mL) using a dual-syringe injector (Stellant, Medrad, Indianola, PA, USA). The contrast dose was tailored to the patient’s body weight comprising a volume of 0.7 mL/kg at a fixed injection duration of 10 s. For the high-pitch spiral mode, the start phase for data acquisition of the most cranial slice was selected at 60% of the R-R interval in all 70 patients. The pitch was set to the maximum of 3.4.

### Image reconstruction and analysis

Image reconstruction was performed using both standard FBP and SAFIRE techniques. SAFIRE applies a noise-modeling technique based on the original raw data^24^. For both FBP and SAFIRE, a sharp reconstruction kernel (B46f and I46f), a section thickness of 0.6 mm, and a reconstruction increment of 0.4 mm were used. All images were transferred to a dedicated multi-modality workstation (Syngo Multi Modality Workplace, Siemens) for further analysis. Subjective image quality and stent assessment were performed by consensus of one cardiologist and one radiologist, both with five years of experience in cardiac CT, who were blinded to the reconstruction technique and CCA results. A final agreement was reached if there were inconsistences between two readers. Images were displayed with a window level and width of 450 and 1,200 HU and were rated using a 5-point Likert scale according to the severity of image noise, quality of contour delineation, and general image impression (1 for poor; 2 for fair; 3 for moderate; 4 for good; 5 for excellent). Stents rated with an image quality score of 1 were deemed non-diagnostic. Disagreements were resolved by consensus, which was achieved in all initially discordant cases.

**Figure 3:**
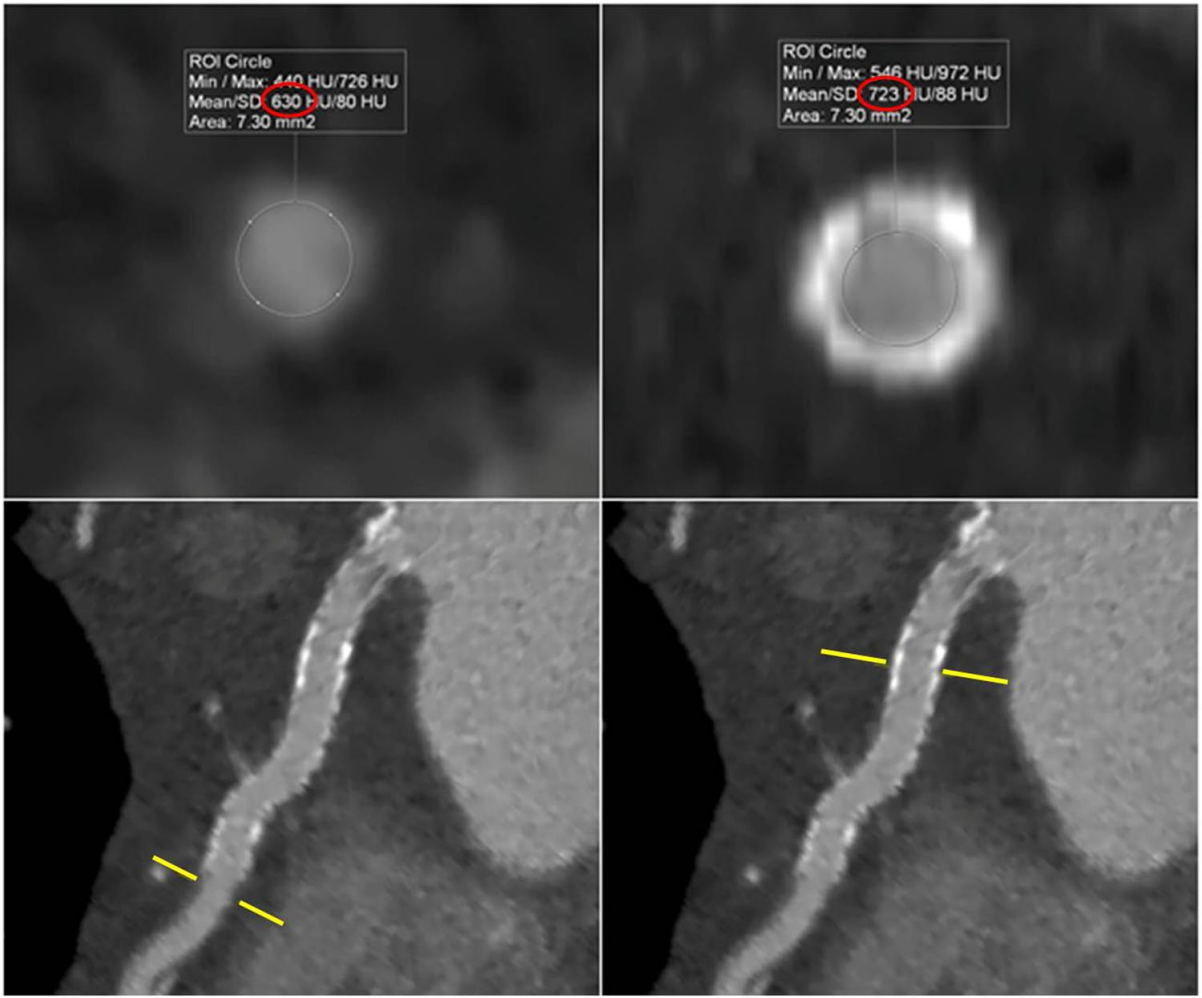
Calculation of stent-lumen attenuation increase ratio (SAIR). SAIR was calculated using the following equation: stent lumen attenuation increase ratio = (in-stent attenuation − coronary lumen attenuation)/coronary lumen attenuation. Example of coronary lumen attenuation (630 HU) as well as in-stent lumen attenuation (723 HU) measured in a sinogram affirmed iterative reconstruction series. SAIR = (723 − 630)HU/630 HU = 0.148.

For each dataset, attenuation values inside the visible stent lumen were measured using a region of interest (ROI) technique. The size of the ROI was drawn as large as possible, but in such a fashion as to exclude stent struts and artefacts. Three measurements per-stent (proximal, middle, distal) as well as three measurements in the same native coronary vessel were performed in identical locations across the reconstruction series. In-stent lumen attenuation and image noise expressed as the standard deviation (SD) of Hounsfield Unit (HU) attenuation were measured. In-stent signal-to-noise ratio (SNR) was calculated(in-stent attenuation/in-stent standard deviation). To assess attenuation effects arising from metallic stent components on the luminal display, the stent lumen attenuation increase ratio (SAIR) was calculated using the following equation: stent lumen attenuation increase ratio = (in-stent attenuation − coronary lumen attenuation)/coronary lumen attenuation**(**Fig. 3).

ISR was defined as lumen reduction ≥50% anywhere within the stent (inner-stent restenosis) or within 5 mm proximal or distal to the stent margins (peri-stent restenosis). All non-diagnostic stents were considered as harboring ISR for statistical analysis.

### Conventional coronary angiography (CCA)

CCA was performed on a cardiac interventional system (AlluraXper® FD20, Philips, Amsterdam, Netherland) with standardized projections and evaluated by one interventional cardiologist with more than five years of experience in coronary angiography. Two experienced operators, blinded to the CT findings, evaluated the stented and peri-stent segments by using the “sharpest and tightest” view of the target lesion free of foreshortening or vessel overlap. The analysis of angiograms was performed with an automated edge-contour QCA system (QAngio XA 7.2.24, Medis Medical Imaging systems, BV, Leiden, Netherlands). The definition of ISR was in analogy to that of DSCT.

### Radiation dose parameters

For CCTA, the dose-length product, defined as total radiation energy absorbed by the patient’s body, was measured in mGy × cm in each patient. The effective radiation dose (ED) was calculated as the dose-length product times a conversion coefficient for the chest(K = 0.014 mSv/mGycm).For CCA, we calculated ED in men and women by multiplying the dose-area product by a conversion factor(K = 0.10 mSv/mGycm^2^) for lateral and posterior-anterior radiation exposure in the chest area.

### Statistical analysis

Statistical analysis was performed by using SPSS 19.0 software (SPSS Inc, Chicago, IL, USA). Continuous variables were expressed as mean ± SD, and discrete variables as absolute numbers and percentages. Student’s t test was used to evaluate differences in continuous variables between two groups, and the Chi-squared test or Fisher’s exact test was used to test differences concerning categorical data.

Evaluability (number of stented segments evaluable/total number of stented segments), sensitivity, specificity, negative predictive value, positive predictive value and The area under ROC curve (AUC) were calculated and compared to CCA in a per-stent analysis, which included only stent segments classified as evaluable. We also performed a stent-based analysis including all stent segments, with non-evaluable segments censored as “positive” for ISR. The 95% confidence interval for these parameters was calculated by using the ratio estimator for variance.

Sensitivity, specificity, positive predictive value (PPV) and negative predictive value (NPV) for the detection of significant ISR were calculated for both SAFIRE and FBP. The diagnostic accuracy of each reconstruction algorithm was calculated with the use of CCA as the reference standard. AUC of SAFIRE and FBP was compared using Hanley & McNeil statistics.

Kappa statistics were performed for inter-observer and intra-observer agreement by reproducibility analysis, which was defined as fair (kappa = 0.21 to 0.40), moderate (kappa = 0.41 to 0.60), good (kappa = 0.61 to 0.80), and excellent (kappa = 0.81 to 1.00).
